# Factors Associated with the Development of Drug Resistance Mutations in HIV-1 Infected Children Failing Protease Inhibitor-Based Antiretroviral Therapy in South Africa

**DOI:** 10.1371/journal.pone.0133452

**Published:** 2015-07-21

**Authors:** Theresa M. Rossouw, Ute D. Feucht, George Melikian, Gisela van Dyk, Winifred Thomas, Nicolette M. du Plessis, Theunis Avenant

**Affiliations:** 1 Institute for Cellular and Molecular Medicine, Department of Immunology, Faculty of Health Sciences, University of Pretoria, Pretoria, South Africa; 2 Department of Paediatrics, Kalafong Provincial Tertiary Hospital, University of Pretoria, Pretoria, South Africa; 3 AIDS Healthcare Foundation, Los Angeles, CA, United States of America; Centro de Biología Molecular Severo Ochoa (CSIC-UAM), SPAIN

## Abstract

**Objective:**

Limited data are available from the developing world on antiretroviral drug resistance in HIV-1 infected children failing protease inhibitor-based antiretroviral therapy, especially in the context of a high tuberculosis burden. We describe the proportion of children with drug resistance mutations after failed protease inhibitor-based antiretroviral therapy as well as associated factors.

**Methods:**

Data from children initiated on protease inhibitor-based antiretroviral therapy with subsequent virological failure referred for genotypic drug resistance testing between 2008 and 2012 were retrospectively analysed. Frequencies of drug resistance mutations were determined and associations with these mutations identified through logistic regression analysis.

**Results:**

The study included 65 young children (median age 16.8 months [IQR 7.8; 23.3]) with mostly advanced clinical disease (88.5% WHO stage 3 or 4 disease), severe malnutrition (median weight-for-age Z-score -2.4 [IQR -3.7;-1.5]; median height-for-age Z-score -3.1 [IQR -4.3;-2.4]), high baseline HIV viral load (median 6.04 log_10_, IQR 5.34;6.47) and frequent tuberculosis co-infection (66%) at antiretroviral therapy initiation. Major protease inhibitor mutations were found in 49% of children and associated with low weight-for-age and height-for-age (p = 0.039; p = 0.05); longer duration of protease inhibitor regimens and virological failure (p = 0.001; p = 0.005); unsuppressed HIV viral load at 12 months of antiretroviral therapy (p = 0.001); tuberculosis treatment at antiretroviral therapy initiation (p = 0.048) and use of ritonavir as single protease inhibitor (p = 0.038). On multivariate analysis, cumulative months on protease inhibitor regimens and use of ritonavir as single protease inhibitor remained significant (p = 0.008; p = 0.033).

**Conclusion:**

Major protease inhibitor resistance mutations were common in this study of HIV-1-infected children, with the timing of tuberculosis treatment and subsequent protease inhibitor dosing strategy proving to be important associated factors. There is an urgent need for safe, effective, and practicable HIV/tuberculosis co-treatment in young children and the optimal timing of treatment, optimal dosing of antiretroviral therapy, and alternative tuberculosis treatment strategies should be urgently addressed.

## Introduction

Treatment of HIV-infected children with antiretroviral therapy (ART) is challenging. Children’s higher HIV-1 viral load (VL), poor tolerability of antiretroviral medication, complex adherence issues and previous exposure to ART through prevention of mother-to-child transmission (PMTCT) strategies potentially contribute to the more rapid development of HIV-1 drug resistance in children compared to adults [1–8]. The situation is further complicated by frequent co-infections, such as tuberculosis (TB), the treatment of which could further impair adherence, increase toxicity and pose the risk of significant drug-drug interactions [9].

Protease inhibitor (PI)-based ART is becoming more important in the management of HIV-1 infected children in the developing world. The CHER trial demonstrated that early treatment of infants with boosted PI-based regimens reduce early infant mortality by 76% and HIV progression by 75% in a developing world setting [10]. PI-based ART is necessitated by the high prevalence of resistance to the non-nucleoside reverse transcriptase inhibitors (NNRTI) in infants 6–8 weeks after PMTCT [11]. Two subsequent trials confirmed that children under the age of three years have a reduced risk of death and ART failure when started on a ritonavir-boosted lopinavir (LPV/r) rather than nevirapine-based regimens, regardless of previous NNRTI exposure [12,13]. The World Health Organization (WHO) now recommends the use of PI-based ART in all children under the age of three years regardless of previous NNRTI exposure through PMTCT and also for all children failing NNRTI-based first-line therapy [14].

The feasibility of large-scale implementation of PI-based regimens in resource-limited settings is, however, questionable. LPV/r is the only co-formulated PI available in a paediatric formulation (Kaletra) in the developing world. LPV/r liquid is expensive, unpalatable and requires refrigeration while LPV/r tablets are relatively large and have uncertain bioavailability when broken [1,2]. Questions also remain about the appropriate weight-adjusted dose, which is most likely higher than in adults, especially when combined with rifampicin-based TB treatment with its potential for drug-drug interactions with ART [15–19]. Limited data exist about the long-term treatment outcomes of children on this regimen in settings with high TB burdens, especially in terms of the development of major PI drug resistance mutations.

This study assessed the proportion of paediatric patients failing PI-based ART in a public sector setting in South Africa–a high TB burden country–with major PI drug resistance mutations and determined factors associated with these mutations.

## Methods

The study population consisted of children who initiated PI-based ART in the Tshwane District, South Africa, and had a genotypic HIV-1 drug resistance test done between 2008 and 2012. ART regimens were prescribed according to national guidelines [20] and consisted of two nucleoside reverse transcriptase inhibitors (NRTIs) and a PI (LPV/r) for children below 3 years of age at ART initiation. Before LPV/r safety data became available, ritonavir as single PI (RTV-sPI) was used between 2004 and 2008 in children younger than 6 months of age and during co-treatment with rifampicin-based TB treatment. HIV physicians switched ART regimens using the national protocol as guideline. Reasons for ART switches included concomitant TB treatment, advancing age (PI changed to efavirenz (EFV) when >3 years of age), side-effects (mostly stavudine (d4T) changed to abacavir (ABC)), or switch to second-line ART due to treatment failure.

Children were assessed monthly while clinically unwell and thereafter 3-monthly. Data collection included demographic and anthropometric measurements, WHO HIV clinical staging and routine blood tests, including CD4+ T-cell counts/ percentages and HIV RNA plasma VL at ART initiation and six-monthly on follow-up. We recorded ART regimens, concomitant TB treatment, LPV/r dose and LPV modifications during TB treatment, with strategies changing over time from use of RTV-sPI to doubling the dose of LPV/r (ddLPV/r) to super-boosted LPV with additional ritonavir (LPV/R^+^).

Virological failure was defined as a HIV-1 VL >40 copies/ml, confirmed on repeat testing by the genotyping laboratory. Duration of virological failure was estimated as the interval, in months, between the first date of viraemia (as defined above) occurring at least 6 months post ART initiation and the date of resistance testing. In cases where virological suppression was never achieved, the duration of failure was calculated as the interval between ART initiation and the date of resistance testing. Time to virological suppression was calculated as the interval between ART initiation and the date of the first episode of virological suppression prior to the date of resistance testing.

Genotypic drug resistance testing was requested in children with virological failure when interventions to improve adherence had failed to result in viral suppression. HIV RNA was quantified in plasma using the bioMéRIEUX Nuclisens EasyQ HIV-1 VL Assay v2.0 (bioMéRIEUX, SA), based on a nucleic acid sequenced based amplification method, according to the manufacturer’s instructions. The Department of Immunology, University of Pretoria, performed population-based genotyping on samples with confirmed VL >1000 copies/ml. RNA was extracted from 200μl of plasma using Nuclisens miniMAG extraction kit (bioMéRIEUX, SA) and reversed transcribed into complementary DNA using the Superscript III kit (Invitrogen Corporation, Carlsbad, CA) and a gene specific primer RT21 [21]. A PCR product, containing 1315-bp, was generated from the HIV-1 subtype C *pol* region. Sequencing was conducted on the HIV-1 protease (99 codons) and the first 300 codons of the reverse transcriptase (RT) gene.

The protease and RT gene regions were amplified from the cDNA using Platinum taq polymerase (Invitrogen Corporation, Carlsbad, CA) and a nested PCR protocol. The second round PCR products were subjected to Agarose gel (1%) electrophoresis and visually compared to a 200bp DNA ladder (Fermentas, Maryland, USA). Amplicons were purified using the PureLink Invitrogen PCR purification kit (Invitrogen Corporation, Carlsbad, CA). Sequencing reactions were performed by means of the Big Dye terminator chemistry (Applied Biosystems Inc., Foster City, CA) using 4 primers and were run on an automated 3500xl, 24 capillary Genetic Analyzer (Applied Biosystems Inc, Forster City, CA).

Sequencing data were assembled and manually edited using CLC DNA Workbench 5.7.1 software (CLC bio, Denmark) and base calling was performed on each chromatogram. Sequences were submitted to the Stanford HIV drug resistance (HIVDB) website (http://hivdb.stanford.edu) to identify HIV-1 drug resistance mutations (according to the ISA_USA 2013 HIV drug resistance mutation list) and to predict drug susceptibility, while the HIV-1 subtype was assigned on the Stanford database using the Rega HIV-1 subtyping tool. Frequencies of mutations were determined and resistance patterns analysed with emphasis on clinical relevance. The nucleotide sequences of these protease and RT genes have been deposited in the GenBank database under accession numbers KT031999-KT032063.

The Research Ethics Committee, Faculty of Health Sciences, University of Pretoria, approved the study protocol (46/2011). The need for informed consent was waived since all tests were part of routine clinical care and all data were entered and analysed anonymously.

## Statistical Analysis

Statistical differences between nominal variables were tested using the Chi-squared test; Fisher’s exact test was used when expected counts were less than 5 per cell. For continuous variables, statistical difference based on the mean was calculated using t-tests when assumptions of normality were satisfied. Normality criteria were determined using Shapiro-Wilk testing and, when not satisfied, the nonparametric Mann-Whitney U test was used to test for differences in central tendency. For binary outcomes, such as the presence of major PI mutations, logistic regression analyses via generalized linear modelling methods were employed, with additional adjustments for duration of exposure of all independent variables (duration of follow-up, time on ART, or duration in virological failure). Differences in time to event variables were measured using Kaplan-Meyer methods. Missing data points were excluded from the analysis.

## Results

The cohort includes 65 children who were initiated on PI-based regimens with a median age of 16.8 months [IQR 7.8; 23.3] at ART initiation and a male-to-female ratio of 1.95. The clinical characteristics of the cohort, as shown in Table 1, reveal the advanced clinical disease, severe malnutrition, high TB burden and high VLs at ART initiation. The median ART duration until genotyping was 41.9 months [IQR 21.0; 56.6].

**Table 1:** Clinical and laboratory findings of the cohort.

Features:	Median [IQR]
**Age and time period (months)** ^1^	
- Age at ART initiation	16.8 [7.8; 23.3]
- Age at genotyping	56.3 [34.8; 81.4]
- Duration of ART	41.9 [21.0; 56.6]
- Duration of PI exposure	25.5 [15.7; 40.4]
- Duration of virological failure*	38.0 [19.1; 51.0]
**Anthropometry (baseline)** ^2,^ **	
- Weight-for-age Z-score	-2.4 [-3.7; -1.5]
- Height-for-age Z-score	-3.1 [-4.3; -2.4]
- BMI-for-age Z-score	-0.6 [-2.4; 0.3]
**CD4 (baseline)** ^3^	
- Absolute CD4 count	558 [251; 899]
- CD4%	13.8 [8.4; 19.7]
**Log_10_ VL (baseline)** ^4^	6.04 [5.34; 6.47]
	**Nr (%)**
**WHO staging (baseline)** ^2^	
- Stage 1	3 (5.0%)
- Stage 2	4 (6.5%)
- Stage 3	21 (34.4%)
- Stage 4	33 (54.1%)
**CD4% in categories (baseline)** ^3^	
- <15%	35 (58.3%)
- 15%-25%	17 (28.3%)
- >25%	8 (13.3%)
**VL in categories (baseline)** ^4^	
- <5 log_10_	8 (13.6%)
- 5–6 log_10_	21 (35.6%)
- ≥6 log_10_	30 (50.8%)
**ART initiation regimen** ^1^	
- d4T + 3TC + LPV/r	42 (64.6%)
- d4T + 3TC + RTV-sPI	- 16 (24.6%)
- d4T + 3TC + LPV/R^+^	3 (4.6%)
- ABC + 3TC + LPV/r	2 (3.1%)
- AZT + 3TC + LPV/r	1 (1.55%)
- AZT + 3TC + RTV-sPI	1 (1.55%)
**ART adherence**	
- Documented ART interruption^2^	42 (68.9%)
**TB treatment** ^2^	
- Ever on TB treatment	47 (77%)
- Never on TB treatment	14 (23%)
**Time on TB treatment** ^5^	
- Prior to ART (completed)	4 (8.5%)
- At ART initiation	31 (66.0%)
- After ART initiation	12 (25.5%)

Data collected from 65^1^, 61^2^, 60^3^, 59^4^ and 47^5^ patients respectively

**Abbreviations:** ART = antiretroviral therapy; PI = protease inhibitor; IQR = interquartile range; BMI = Body mass index; CD4 = CD4+ T-cell count in cells/mm^3^; CD4% = CD4 percentage; VL = HIV viral load in log_10_ copies/ml; Nr = number; WHO stage = HIV disease staging according to World Health Organization; d4T = stavudine; 3TC = lamivudine; LPV/r = boosted lopinavir; RTV-sPI = ritonavir as full-dose single protease inhibitor; LPV/R^+^ = super-boosted lopinavir; ABC = abacavir; AZT = zidovudine; TB = tuberculosis.

*From first failure time point (using LDL cut-off & >6 months on ART) to date of genotype

**Age- and sex-adjusted anthropometry according to WHO growth standards [22]

Of the 61 children with reliable TB data, 77% had received co-treatment for TB and HIV disease; 66% of whom were on TB treatment at the time of ART initiation. TB treatment was started at a median of 42 days before ART initiation [IQR: 84 days prior; 18 days post ART initiation]. The majority of the children (64.6%) were initiated on a combination of d4T, lamivudine (3TC) and LPV/r, while the second most common regimen was d4T, 3TC and RTV-sPI (24.6%). At time of genotyping, 44 patients were still on a PI-based regimen [cumulative months on PI 28.3 [IQR 20.0; 46.6], while 19 had been switched to NNRTI-based regimens and had not been on a PI for a median of 25.5 months [IQR 15.6; 40.4]. One patient was on both a PI and NNRTI and one was not on any ART. The majority were still on d4T (76.9%) and 3TC (90.8%), while 13.8% were now on ABC, 10.8% on didanosine (ddI) and 7.7% on zidovudine (AZT).

The median VL log_10_ at time of genotyping was 4.15 [IQR 3.50; 5.01]. Genotypic drug resistance testing was successful in all 65 patients. All viruses belonged to HIV-1 subtype C and had at least one drug resistance mutation. All but two of the genotypes (97%) had NRTI mutations and, as expected, the M184V mutation, which confers resistance to 3TC/emtricitabine, was the most common, occurring in 89%. Thymidine analogue mutations (TAMs) were found in 25%, with half (50%) of these genotypes showing multiple (≥3) TAMs and the majority (71% of total TAMs) following the TAM2 pathway. The K65R mutation was rare in this patient population, occurring in only 3%. Furthermore, evidence of resistance to NNRTIs was found in 45% genotypes and the most common NNRTI mutations were K103N (23%), P225H and Y188C (9% each). NNRTI mutations predictably occurred more commonly in patients on an NNRTI at the time of genotype (67.9% versus 32.1%).

**Fig 1:**
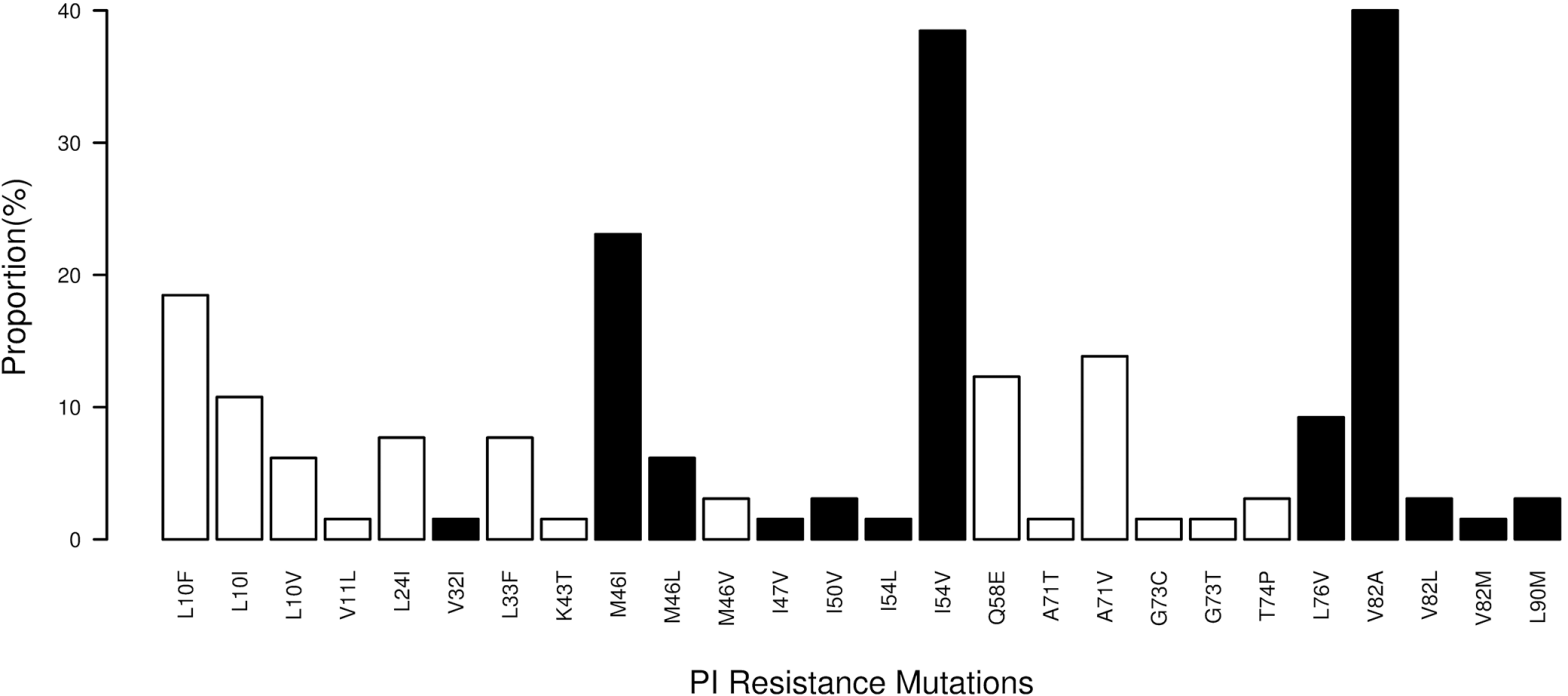
Protease inhibitor (PI) drug resistance mutations (black bars denote major PI mutations, white bars denote minor PI mutations).

Thirty-two of 65 (49%) genotypes had major and 51% minor PI mutations (Fig 1) with the most common major mutations being V82A (40%), I54V (38.5%), M46I (23%) and L76V (9.2%). Among patients with at least one major PI mutation (n = 32), the median number of major PI mutations was 3 (11 with 1 or 2 mutations, 13 with 3 mutations, and 8 with 4 or 5 mutations) and the median number of minor PI mutations was 1 [IQR 1; 2]. There was no difference in the prevalence of major PI mutations regardless of whether the child was still on a PI-regimen or had been switched to an NNRTI-regimen at the time of genotyping (p = 0.98).

Univariate analysis (Table 2) showed that the patients with major PI mutations were younger at ART initiation than those without mutations, but this was not statistically significant (p = 0.18). Both weight-for-age and height-for-age Z-scores were significantly associated with developing PI mutations (p = 0.039; p = 0.05), as were longer duration on ART, time on PI, and duration of virological failure (p = 0.003, p = 0.001 and p = 0.005 respectively). Baseline clinical staging (p = 0.80), CD4 count/percentage (p = 0.85; p = 0.74) and baseline VL (p = 0.35) were not associated with PI mutations, although children in the highest two VL quartiles took longer to reach virological suppression (p = 0.008).

**Table 2:** Univariate analysis comparing patients with and without major protease inhibitor (PI) drug resistance mutations.

Features:	With major PI mutations	Without major PI mutations	
	(Median [IQR])	(Median [IQR])	
	n = 32	n = 33	p value
**Age and time period (months)** ^1^			
- Age at ART initiation	11.4 [7.6; 19.1]	17.3 [7.9; 28.0]	0.18
- Duration of ART	50.2 [33.2; 64.7]	25.5 [18.4; 48.2]	0.003
- Duration of PI exposure	34.1 [25.1; 51.2]	20.7 [15.2; 25.5]	0.001
- Duration of virological failure*	45.0 [28.4; 59.5]	22.1 [16.8; 47.6]	0.005
**Anthropometry (baseline)** ^2,^ **			
- Weight-for-age Z-score	-3.0 [-3.7; -1.9]	-2.0 [-3,5; -0.9]	0.039
- Height- for-age Z-score	-3.2 [-4.6; -2.7]	-3.0 [-3.5; -1.8]	0.05
- BMI-for-age Z-score	-0.7 [-2.5; 0.1]	-0.6 [-2.2; 0.7]	0.45
**CD4 (baseline)** ^3^			
- Absolute CD4 count	560 [192; 911]	548 [281; 828]	0.85
- CD4%	13.7 [8.1; 20.1]	14.5 [10.8; 19.5]	0.74
**Log_10_ VL (baseline)** ^4^	5.98 [5.55; 6.48]	5.91 [5.18; 6.38]	0.35
**HIV VL suppression**			
- Months to LDL (if suppressed)^5^	15.1 [12.4; 27.0]	6.5 [4.6; 8.6]	0.014^**#**^
**Associated mutations**			
- Nr of minor PI mutations^1^	1.0 [1.0; 2.0]	0.0 [0.0; 0.0]	<0.001
	Nr (%)	Nr (%)	
**VL in categories (baseline)** ^4^			
- <5 log_10_	2 (6.9%)	5 (16.7%)	
- 5–6 log_10_	11 (37.9%)	10 (33.3%)	0.612
- ≥6 log_10_	16 (55.2%)	15 (50.0%)	
**HIV VL suppression**			
- VL ever LDL (‘undetectable’)^1^	15 (47%)	13 (39%)	0.72
- LDL by 12 months on ART^5^	3 (20.0%)	11 (84.6%)	0.001
**TB treatment**			
- On TB treatment while on ART^2^	25 (83.3%)	18 (58.1%)	0.036
**Time on TB treatment** ^2^			
- Never/prior to ART (completed)	5 (16.7%)	13 (42.0%)	Ref
- At ART initiation	18 (60.0%)	13 (41.9%)	0.048
- After ART initiation	7 (23.3%)	5 (16.1%)	0.12
**PI use during TB co-treatment**			
- Ever on RTV-sPI ^6^	18 (72.0%)	7 (38.9%)	0.038
- Ever on ddLPV/r ^7^	10 (41.7%)	8 (50.0%)	0.85
- Ever on LPV/R^+ 8^	2 (8.3%)	3 (16.7%)	0.46
**Associated mutations** ^9^			
- Any NRTI mutations	31 (100%)	31 (93.9%)	0.49
- Any TAMs	11 (35.5%)	5 (15.2%)	0.07
- Any NNRTI mutations	15 (48.4%)	14(42.4%)	0.82

Data collected from 65^1^, 61^2^, 60^3^, 59^4^, 28^5^, 43^6^, 40^7^, 42^8^ and 64^9^ patients respectively

**Abbreviations:** ART = antiretroviral therapy; PI = protease inhibitors; IQR = interquartile range; BMI = body mass index; CD4 = CD4+ T-cell count in cells/mm^3^; CD4% = CD4 percentage; VL = HIV viral load; LDL = lower than detectable level; Nr = number; TB = tuberculosis; Ref = reference value; RTV-sPI = ritonavir as full-dose single protease inhibitor; ddLPV/r = double-dose boosted lopinavir; LPV/R^+^ = super-boosted lopinavir; NRTI = nucleoside reverse transcriptase inhibitor; TAMs = thymidine analogue mutations; NNRTI = non-nucleoside reverse transcriptase inhibitor

*From first failure time point (using LDL cut-off & >6 months on ART) to date of genotype

**Age- and sex-adjusted anthropometry according to WHO growth standards [22]

#Corresponding p-value for VL<1000 = 0.036

There was no difference in the number of children in each group who ever had viral suppression (39% vs. 47%, p = 0.72). Among patients who achieved virological suppression to lower than detectable levels (LDL) (n = 28), 57% were suppressed at a single visit, 14% at 2 visits, and the remainder (29%) at ≥3 visits. Time to a first episode of suppression to LDL was significantly longer for patients with at least one major PI mutation (median 15.1 months vs. 6.5 months, p = 0.014), while failure to suppress to LDL by 12 months was associated with a higher risk of developing PI mutations (p = 0.001). While the VL at genotype was equivalent between children with and without major PI mutations (p = 0.35), patients with ≥2 major PI mutations had a significantly higher VL at time of genotype than those with ≤1 major PI mutation (log_10_ VL 4.45 vs 3.79; p = 0.021). There was no difference in clinically documented ART interruptions (~69% in both groups; p = 1).

Co-treatment for TB and HIV significantly increased the risk for major PI mutations (p = 0.036), with children on TB treatment at the time of ART initiation significantly more likely to develop PI mutations (p = 0.048). There was no difference between the groups if TB treatment had been started later while on PI (p = 0.12). After adjusting for baseline anthropometry, TB disease at ART initiation and malnutrition were found to be independently associated with the development of PI mutations [TB odds ratio (OR) 4.6; 95% CI 1.1 to 22.3; p = 0.04] and this association held even after further adjustment for total time on PI.

**Fig 2:**
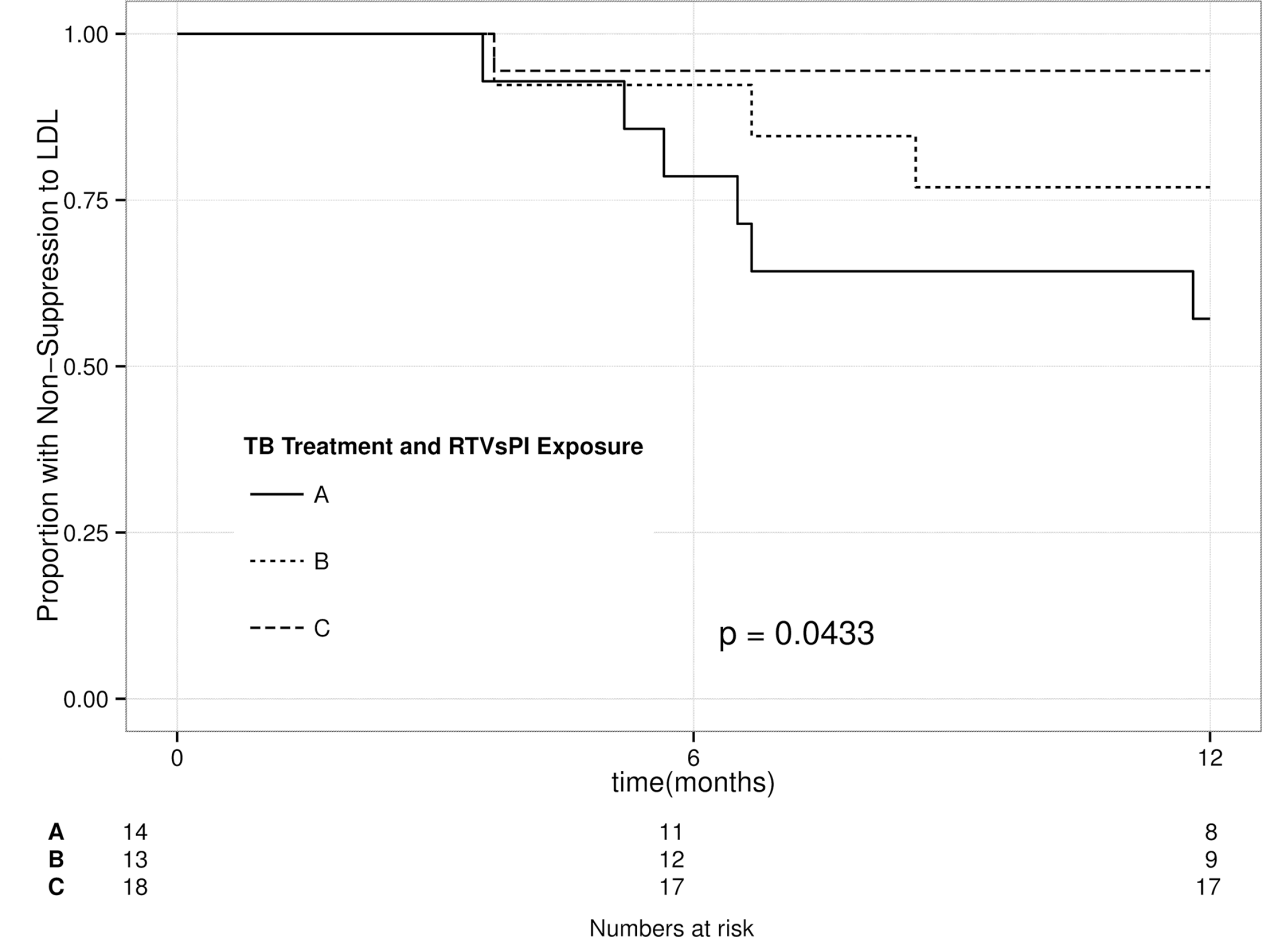
Kaplan-Meier curve of time to virological suppression among children with or without use of ritonavir as single protease inhibitor (RTV-sPI) at the time of protease inhibitor (PI) initiation. Groups: (A) No RTV-sPI exposure or history of exposure to TB therapy; (B) No RTV-sPI exposure, on TB treatment at time of PI initiation; (C) History of RTV-sPI exposure, and on TB treatment at time of PI initiation.

Analysis of different PI dosing strategies showed an association between children on RTV-sPI during the period of co-treatment of TB and the development of PI mutations (p = 0.038). Among the 39 children with no RTV-sPI exposure there was a trend for risk of major PI mutation if they also received TB treatment; however, the numbers were small and did not reach statistical significance OR = 2.0 [95% CI 0.5 to 9.8]. There was no evidence of an association between the use of either ddLPV/r or super-boosted LPV/R^+^ and the development of PI mutations. There was no evidence of confounding or co-linearity between failure to achieve virological suppression and exposure to RTV-sPI, suggesting that both were independently associated with the development of major PI resistance mutations. However, restricting the analysis to only children who had been on TB treatment at the time of ART initiation showed that patients on RTV-sPI were less likely to achieve virological suppression by 12 months (OR = 12.8; 95% CI 1.8 to 263.0; p = 0.028) and took longer to achieve such suppression (p = 0.043) (Fig 2). Multivariate analysis controlled for possible confounding revealed that only duration of RTV-sPI and duration of PI exposure predicted the development of major PI mutations (p = 0.033 and p = 0.008 respectively).

Table 3 shows the projected susceptibility of children with and without major PI mutations to the three major available ART classes. There was a significant difference in terms of susceptibility to the PIs, with children with major PI mutations more likely to have high-level resistance to LPV/r and intermediate-level resistance to ritonavir-boosted tipranavir and darunavir (p<0.001). There was, however, no difference between the groups in respect of susceptibility to NRTIs and NNRTIs.

**Table 3:** Comparison of projected susceptibility to antiretroviral medication in children with and without major protease inhibitor (PI) drug resistance mutations.

Projected susceptibility to antiretroviral drug classes:			With major PI mutations	Without major PI mutations	
			Nr (%)	Nr (%)	
			n = 32	n = 33	p value
PI:^1^	LPV/r:	Susceptible	0 (0%)	33 (100%)	
		Intermediate	12 (37.5%)	0 (0%)	**<0.001**
		High	20 (62.5%)	0 (0%)	
	DRV/r:	Susceptible	22 (68.8%)	33 (100%)	
		Intermediate	10 (31.2%)	0 (0%)	**<0.001**
	TPV/r:	Susceptible	5 (15.6%)	32 (97.0%)	
		Intermediate	27 (84.4%)	1 (3.0%)	**<0.001**
NNRTI:^2^	EFV:	Susceptible	16 (51.6%)	19 (57.6%)	
		Intermediate	2 (6.5%)	1 (3.0%)	0.77
		High	13 (41.9%)	13 (39.4%)	
	ETR:	Susceptible	21 (67.7%)	23 (69.7%)	
		Intermediate	10 (32.3%)	10 (30.3%)	1
NRTI:^2^	ABC:	Susceptible	0 (0%)	2 (6.05%)	
		Intermediate	26 (83.9%)	29 (87.9%)	0.22
		High	5 (16.1%)	2 (6.05%)	
	AZT:	Susceptible	22 (71.0%)	29 (87.9%)	
		Intermediate	5 (16.1%)	3 (9.1%)	0.22
		High	4 (12.9%)	1 (3.0%)	
	d4T:	Susceptible	20 (64.5%)	29 (87.9%)	
		Intermediate	6 (19.4%)	3 (9.1%)	0.08
		High	5 (16.1%)	1 (3.0%)	
	TDF:	Susceptible	22 (71.0%)	30 (90.9%)	
		Intermediate	7 (22.6%)	3 (9.1%)	0.08
		High	2 (6.4%)	0 (0%)	
	3TC:	Susceptible	2 (6.45%)	3 (9.1%)	
		Intermediate	2 (6.45%)	0 (0%)	0.42
		High	27 (87.1%)	30 (90.9%)	

Data collected from 65^1^ and 64^2^ patients respectively

Predicted drug susceptibility categories were determined through the Stanford HIVdb Algorithm

**Abbreviations:** PI = protease inhibitors; LPV/r = ritonavir-boosted lopinavir; DRV/r = ritonavir-boosted darunavir; TPV/r = ritonavir-boosted tipranavir; NNRTI = non-nucleoside reverse transcriptase inhibitor; EFV = efavirenz; ETR = etravirine; NRTI = nucleoside reverse transcriptase inhibitor; ABC = abacavir; AZT = zidovudine; d4T = stavudine; TDF = tenofovir; 3TC = lamivudine

## Discussion

This study of children failing ART shows that all children who had genotypic drug resistance testing had at least one HIV-associated drug resistance mutation and that resistance patterns were generally severe. In contrast to previous studies [1,23–31], almost half of the children had major PI mutations and a quarter had TAMs (half of which had multiple TAMs). The duration of treatment exposure may explain some of this difference with our study and others [32–35] showing that cumulative months on PI predisposed patients to the development of resistance.

Even though some studies have shown that age is a risk factor for treatment failure [36–39] and drug resistance [40,41], most likely due to developing metabolic pathways, variable pharmacokinetics and lack of adequate paediatric dosing information, the CHER study did not support this association [42]. While children who developed major PI mutations in our study were younger at the time of ART initiation than those who did not develop mutations, this difference was not statistically significant. However, few children in our study started ART very early.

Malnutrition increases hospitalisation and mortality in HIV-infected children [43–45], but few associations with immunological failure exist [39,46] and none with virological failure on ART [39,47]. There further appears to be no literature about toxicity, other adverse effects, or the ART regimen of choice in severely malnourished children [48] and very few studies have assessed the effect of pharmacokinetics of ART specifically in malnourished children [49,50]. In our study, both weight-for-age and height-for-age Z-scores were significantly associated with developing PI mutations and this association was independent of TB therapy. Malnutrition is characterised by changes in body composition and metabolic function, which may influence the efficacy of ART, and malnourished children are more susceptible to chronic diarrhoea, possibly impeding the absorption of ART [48,51,52].

High pre-initiation VL and/or low CD4 count have been associated with a lower probability of durable viral suppression during PI-based treatment [53–55] as well as with the development of drug resistance to NNRTI-based regimens [40,56]. Our study did not show this, but the majority of children had advanced disease at the time of ART initiation, which complicated comparison. A similar number of children in the two groups achieved virological suppression but, among children who did suppress, those who developed at least one major PI mutation took longer to suppress their VL and were less likely to have achieved suppression by 12 months of ART.

An unexpectedly slow virological response after treatment initiation may occur due to drug intolerance, toxicity, inadequate absorption, resistance, non-adherence and possibly some unknown factors, and such patients should be assessed for adherence to therapy, and if available, drug levels and the presence of resistance [57]. Primary drug resistance is unlikely in our study since very low levels of primary resistance, especially to the NRTIs and PIs, have been reported in South Africa, but no paediatric data are available [21]. Our data also showed no difference in clinically documented treatment interruptions between the groups, although more subtle or objective differences in adherence could not be assessed.

Slow and incomplete virological suppression was, however, associated with the timing of TB co-treatment and the type of PI boosting strategy used. Children who initiated ART while on TB treatment were almost 5 times more likely to develop major PI mutations than children without TB or who had completed TB treatment prior to ART initiation. Several studies have demonstrated worse virological outcomes in children who received TB co-treatment at the time of ART initiation, but this had not been linked to the presence of drug resistance [16,58–60]. TB co-treatment may predispose patients to drug resistance since the large pill burden may impair adherence and rifampicin-induced cytochrome P450 activity may cause sub-therapeutic levels of LPV [9,61,62]. Various strategies have been proposed to overcome this, such as RTV-sPI, addition of more ritonavir to LPV/r (LPV/R+) and doubling the LPV/r dose (ddLPV/r).

RTV, when used as a single PI, is not significantly affected by concurrent rifampicin treatment and was used for co-treatment until data on LPV boosting strategies in children became available [63]. Our study confirms that initiation of ART with RTV-sPI during the period of co-treatment is associated with the development of PI mutations [63–66]. However, not only patients on RTV-sPI developed PI mutations and not all patients initiated on RTV-sPI fail treatment [65]. Our study was too small to allow comparison between LPV/R+ and ddLPV/r, but studies have shown that ddLPV/r results in substantial reductions of LPV concentrations and should not be used in young children [16,17]. While LPV/R+ achieves acceptable LPV concentrations in >85% of children [67,68] it does not completely counteract rifampicin induction of lopinavir in very young children and has been complicated by concerns about significant hepatotoxicity in healthy adult volunteers [61,69]. Zhang et al [70] proposed that an 8-hourly dosing regimen with LPV/r might maintain therapeutic levels of LPV during TB treatment, but this strategy has not yet been assessed in clinical trials.

Modifying TB treatment, by excluding a rifamycin, is an option but may be sub-optimal in HIV-infected patients with active TB and is currently not recommended [71]. Rifabutin is a less potent inducer of CYP3A4 and has little, if any, effect on the serum concentrations of ritonavir-boosted protease-inhibitors and has been used in HIV-TB co-infected adults [72]. Use in children is, however, limited by scanty data, high rates of neutropenia, and the absence of an established safe and effective dose [73].

Major PI mutations have been associated with adverse clinical outcomes. Children with a lopinavir mutation score of ≥6 are less likely to achieve an undetectable VL and to remain virally suppressed [74]. In our study almost 70% of children with major PI mutations were still fully susceptible to darunavir and at least one NRTI (mostly AZT and tenofovir), implying that locally available ‘third-line’ treatment should still be effective. These results support observations that treatment failure on LPV/r safeguards other PIs for future use [30–32,34].

Notwithstanding the lack of validated adherence tools in children, our study is limited by the absence of more objective measures of adherence such as pill counts and LPV levels in hair. Other limitations include incomplete data on PMTCT exposure, no denominator data and the absence of drug resistance testing at ART initiation. In addition, the retrospective nature of this study limited the results to testing of associations and not causation. Nevertheless, our study contains significant strengths, such as the relatively large study population, uniform and simple treatment regimens, and robust clinical data.

## Conclusions

This study identified children at high risk of developing major PI drug resistance mutations. Children on TB treatment at ART initiation, suboptimal ART regimens, malnutrition, and an unexpectedly slow rate of initial viral suppression should be identified and targeted for more intensive monitoring and adherence support. In addition, even though use of RTV-sPI is no longer recommended, children with previous exposure to such a regimen are at high risk of failure and should be provided with frequent VL monitoring and early access to drug resistance testing. Further research into ART dosing in very young and malnourished children should be a priority. Finally, there is an urgent need for safe, effective, and practicable HIV/TB treatment strategies in young children with emphasis on the optimal timing and dosing of ART and potentially alternative TB treatment strategies.
